# The Pharmacological Effects of Lutein and Zeaxanthin on Visual Disorders and Cognition Diseases

**DOI:** 10.3390/molecules22040610

**Published:** 2017-04-20

**Authors:** Yu-Ping Jia, Lei Sun, He-Shui Yu, Li-Peng Liang, Wei Li, Hui Ding, Xin-Bo Song, Li-Juan Zhang

**Affiliations:** 1 College of Pharmaceutical Engineering of Traditional Chinese Medicine, Tianjin University of Traditional Chinese Medicine, Tianjin 300193, China; 15692232675@163.com (Y.-P.J.); sunleitj2013@163.com (L.S.); hs_yu08@163.com (H.-S.Y.); 15692232573@163.com (L.-P.L.); cheercathy@163.com (W.L.); songxinbo@tjutcm.edu.cn (X.-B.S.); 2 Tianjin Zhongyi Pharmaceutical Co., Ltd., Tianjin 300193, China; dinghui.hn@163.com

**Keywords:** lutein, zeaxanthin, carotenoids, AMD, ARC, cataract, cognition, ADI

## Abstract

Lutein (L) and zeaxanthin (Z) are dietary carotenoids derived from dark green leafy vegetables, orange and yellow fruits that form the macular pigment of the human eyes. It was hypothesized that they protect against visual disorders and cognition diseases, such as age-related macular degeneration (AMD), age-related cataract (ARC), cognition diseases, ischemic/hypoxia induced retinopathy, light damage of the retina, retinitis pigmentosa, retinal detachment, uveitis and diabetic retinopathy. The mechanism by which they are involved in the prevention of eye diseases may be due their physical blue light filtration properties and local antioxidant activity. In addition to their protective roles against light-induced oxidative damage, there are increasing evidences that L and Z may also improve normal ocular function by enhancing contrast sensitivity and by reducing glare disability. Surveys about L and Z supplementation have indicated that moderate intakes of L and Z are associated with decreased AMD risk and less visual impairment. Furthermore, this review discusses the appropriate consumption quantities, the consumption safety of L, side effects and future research directions.

## 1. Introduction

Carotenoids can be divided into two main classes: carotenes and xanthophylls. Carotenes are non-polar molecules, which contain only carbon and hydrogen atoms, while xanthophylls are polar carotenoids, which contain at least one oxygen atom [1]. In addition, xanthophylls can be subdivided into hydroxycarotenoids containing one or two hydroxyl groups and ketocarotenoids containing ketone groups. Over 600 types of carotenoids are found in Nature, of which 30–50 are part of the normal human diet. However, only 10–15 are routinely detectable in the human serum, including lutein (L), zeaxanthin (Z) and their metabolites [2,3,4,5]. L and Z are dietary carotenoids derived from dark green leafy vegetables, oranges, yellow fruits and vegetables that form the macular pigment of human eyes [6]. They cannot be synthesized in mammals and must be obtained from the diet for distribution to various tissues, especially the retina [7]. Previous studies have shown that macular pigments (MPs) are related to many static indicators of visual performance, such as visibility, disability glare and the critical flicker fusion threshold (CFF) [8]. In addition, MPs may help to protect against age-related macular degeneration (AMD) because of their effect on blue light filtration and local antioxidant activity [9]. MPs also have anti-inflammatory and light-screening properties [7,9]. As the important components of the MPs, L and Z are selectively concentrated in the foveal region [10,11] where they account for the characteristic central yellow coloration known as the primate macula lutea. Their concentrations are almost 1 mM in the Henle fiber layer which corresponds to the axons of the foveal photoreceptors.

Z dominates the center region, while L is dominant in the peripheral region of the retina [12]. The concentration of the macular pigment drops nearly 100 fold within just 1–2 mm from the foveal center [13]. Another carotenoid, meso-zeaxathin (MZ), which is a stereoisomer of Z, is converted from L within the retina [1]. The position of the double bond in one of the rings in L and Z molecules creates differences in the distribution of these two macular pigments in the retina. In the inner macula, the concentration of Z is about twice that of L. As eccentricity from the fovea increases, the ratio of concentrations continuously changes, with L becoming the dominant component in the peripheral retina. At distances exceeding 6 mm from the fovea, the L:Z ratio is between 2:1 and 3:1 [14,15]. Their antioxidant properties are due to their abilities to quench singlet oxygen, scavenge superoxide and hydroxyl radicals, to protect membrane phospholipids against UV-induced peroxidation, and to reduce lipofuscin formation. In addition, the maximum absorption of these MP is about 450 nm, which is consistent with the action spectrum for light-induced damage [16]. Moreover, L and Z were incorporated in higher amounts into cell membranes in a single orientation, making them behave like optical filters to some extent. Therefore these MP could absorb and attenuate the photic damage in the human lens [17]. In the inner retina they may serve as a filter for high energy and short wave-length blue light [18,19], which may protect the outer retina from photochemical injury induced by these high energy wavelengths [20]. Although blue light has a high energy, blue light filtration may be one of the functions of MP [21]. L and Z are also found in the rod and presumably cone outer segments. While in the outer retina, they may serve as antioxidants. Photoreceptor outer segments contain chromophores that act as photosensitizers susceptible to oxidative damage. L and Z are capable of quenching reactive oxygen species produced from chromophore irradiation, which may protect the retina from the deleterious effects of lipid peroxidation [14].

It is generally assumed that the presence of L and Z is not incidental; rather, such high accumulation in functional areas (like the fovea) implies that L and Z may have some functions [22]. There are three major hypotheses for function of L and Z are commonly proposed, i.e., the acuity, visibility, and protective hypotheses [16,23]. These hypotheses are all based on just two fundamental characteristics of the MP, i.e., their light filtration and antioxidant characteristics.

The eye is a major sensory organ that requires special care for a healthy and productive lifestyle. Numerous studies have identified L and Z to be important components for eye health. Their role in human health, in particular the health of eyes, is established from epidemiological, clinical and interventional studies [5,8]. They constitute the main MPs found in the yellow spot of the human retina which protect the macula from damage by blue light, improve visual acuity and scavenge harmful reactive oxygen species. They have also been linked with reducing the risk of AMD and ARC. They may also enhance visual performance by decreasing chromatic aberration and enhancing contrast sensitivity [24]. Besides, research over the past decade mainly focused on the development of carotenoid-rich foods to boost their intake especially for the elderly population [25,26,27].

In the human retina, the concentration of carotenoids reaches a level between 0.1 and 1 mM in the central fovea [28], which is about 1000 times higher than in other tissues. Both xanthophylls are accumulated in the region of photoreceptor axons [29] and within photoreceptor outer segments (POSs) [30]. Although, macular xanthophylls in POS constitute about 10–25% of the amount in the entire retina [30], the local concentration of macular xanthophylls in membranes of the rod outer segment is about 70% higher than in residual retina membranes [30]. Moreover, Muller cells have also been suggested as a place for xanthophyll accumulation [31].

Although L and Z differ only by the placement of a single double bond, this small change in configuration has a great impact on the function of these two carotenoids [32]. Compared to L, Z is a much more effective antioxidant [33]. MZ also has a greater capability of quenching oxygen radicals than L [13]. The functional differences of these carotenoids correlate with the spatial distribution of L, MZ, and Z. The ratio of L to Z varies linearly with the ratio of rods to cones in the fovea. MZ and Z predominate where cone density is highest and risk of oxidative damage is greatest [33]. The macular pigments also differ in other aspects. For example, L has a greater filtering efficiency, and Z is superior in preventing lipid peroxidation induced by UV light [34]. These essential functions of macular pigment may decrease oxidative stress in the retina and enhance vision in both normal and diseased retinas. Although the structure and properties of L and Z to carotenoids are similar, there are special properties for L and Z because of the oxygen in their structure. It is these special properties that account for the biological functions of L and Z [35]. Nevertheless, there are negative effects of L and Z. For example, increased risk of lung cancer in smokers after L and Z supplementation and the development of crystalline maculopathy after supplementation of high dosages of L and Z [36]. Besides, continuous intake of high doses of L may result in skin yellowing [37]. In this review, the pharmacological effects and mechanisms of L and Z on visual disorders and cognition diseases are summarized. Also, the safe dosages of L and Z as well as their structure-activity relationships are well illustrated. In addition, areas requiring further research on L and Z are also introduced.

## 2. The Structure and Distribution of L and Z

**Figure 1:**
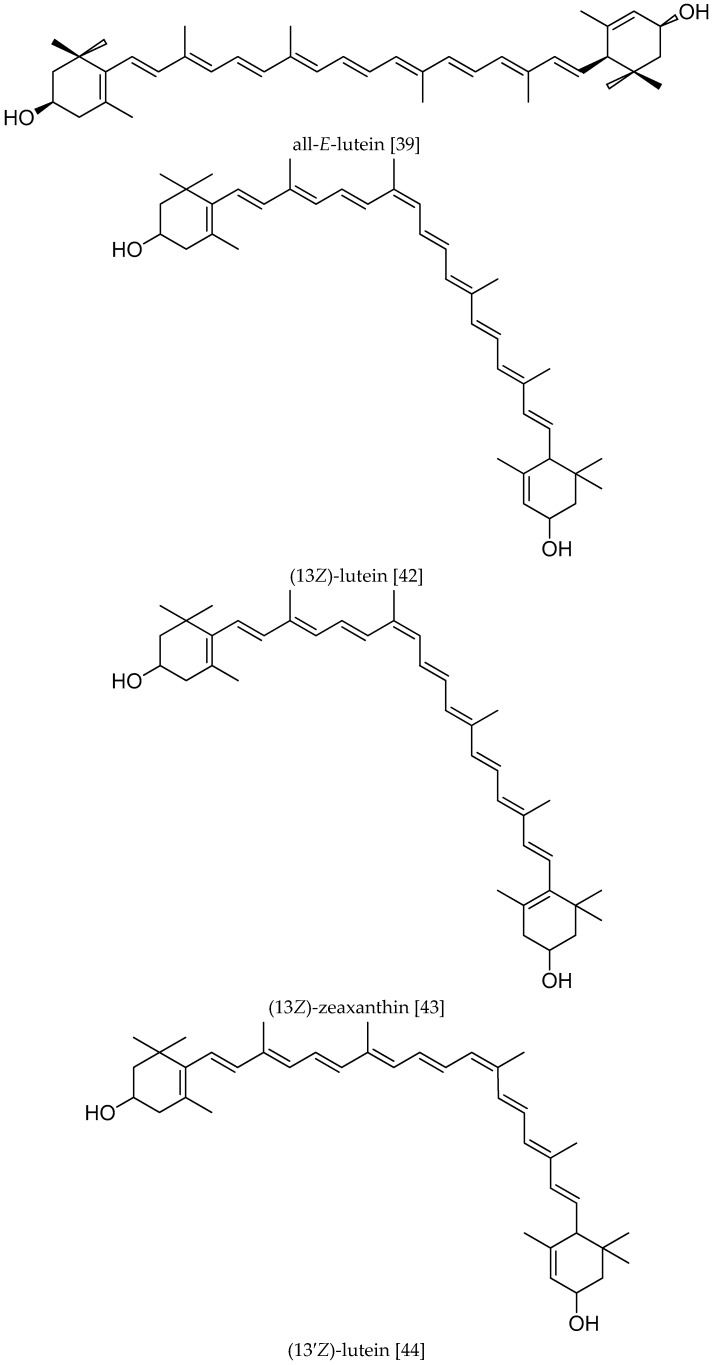

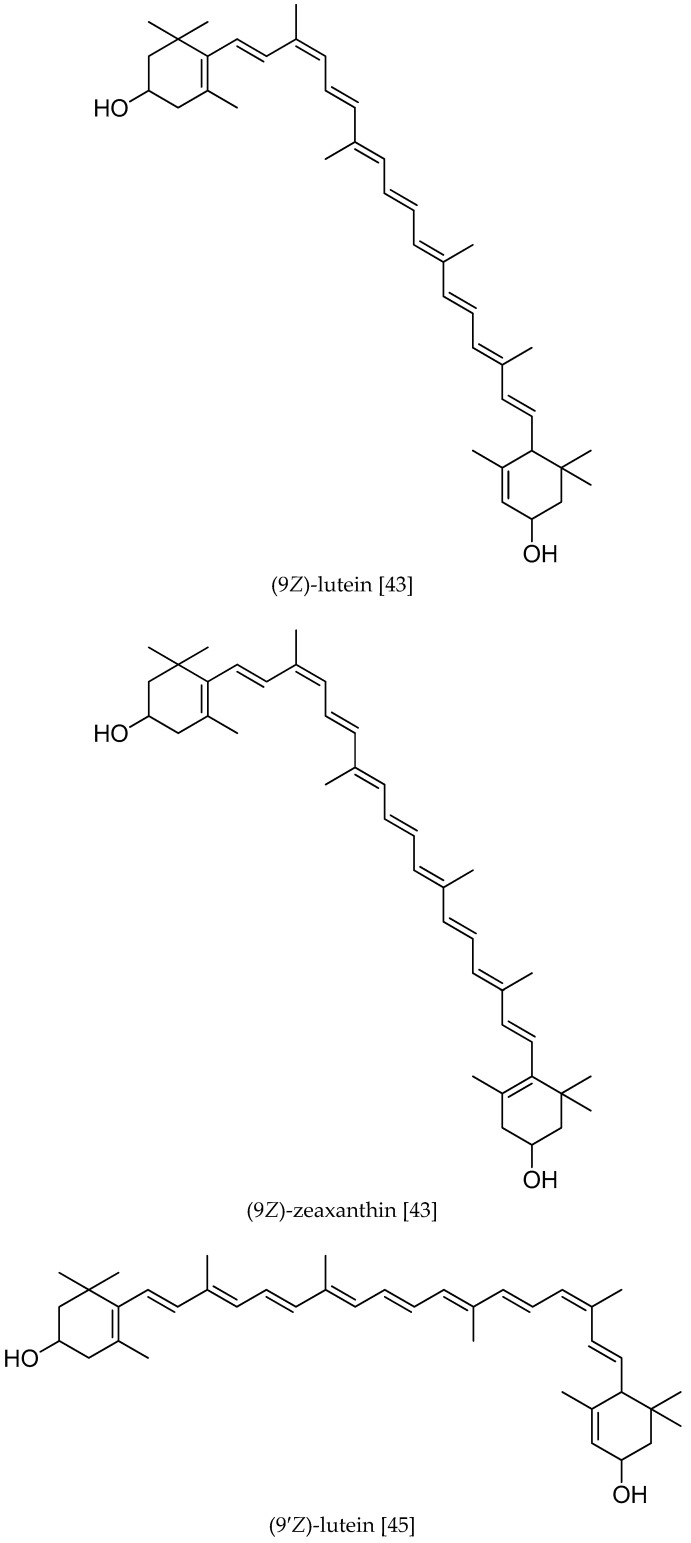

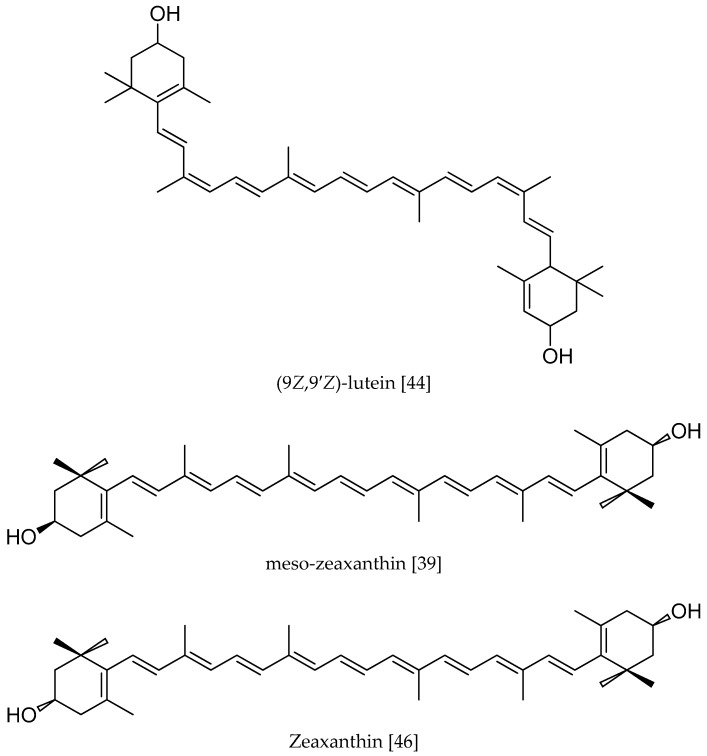
The structures of L, Z and their stereoisomers.

L and Z are relatively polar carotenoids pigments found at high levels in parsley, spinach, kale, egg yolk and lutein-fortified foods. Snodderly [38] demonstrated several beneficial health effects due to their ability to act as scavengers for reactive oxygen species and to bind with physiological proteins in humans. In general, carotenoids are tetraterpenoids with 40 carbon skeleton made up of eight isoprene units and comprising two classes, namely the carotenes (purely unsaturated hydrocarbons) and carotenoids with oxygen atoms, which are referred to as oxygenated carotenoids or xanthophyll carotenoids. The macular carotenoids are dietary L and Z, and their conversion isomer MZ, which are non-provitamin A carotenoids, (i.e., they cannot be converted into vitamin A). Important members of the oxygenated carotenoids are L, Z, β-cryptoxanthin, capsanthin, astaxanthin, and fucoxanthin. The percentages of main carotenoids in human serum are L (20%), lycopene (20%), β-carotene (10%); β-cryptoxanthin (8%), α-carotene (6%) and Z (3%) [4]. L and Z are the main dietary carotenoids found in the human retina [39] and they probably protect the macula from being damaged by blue light, improve visual acuity and scavenge harmful reactive oxygen species [17,18,19,20,21]. L and Z, along with their common metabolite MZ, are commonly referred to as the macular pigments (MPs) [14]. The ratio between L, Z and MZ changes as the eccentricity moves away from fovea [12,40]. Although L and Z were also detected in prenatal eyes, they did not form visible yellow spots. No age-related (between the ages of 3 and 95 years) differences were observed in the quantity of L and Z [40], but the ratio of L to Z differed between infants and adults. In infants, L predominates over Z in the fovea, and the opposite is true after 3 years of age [40,41]. Structurally the difference between L and Z is in the type of ionone ring. L contains a β-ionone ring and an ε-ionone ring, whereas Z has two β-ionone rings. L and Z are isomers, but not stereoisomers, which differ in the location of a double bond unsaturation in the end ring. L can exist in eight possible stereoisomeric forms because of its three chiral centers, but in Nature it exists mainly in the *Z* (*cis*)-form (*R,R,R*). Z, on the other hand, has two chiral centers but, because of symmetry exists in only three stereoisomeric forms: (*R,R*), (*S,S*) and (*R,S*-meso). Although plants and microorganisms can synthesize and interconvert carotenes and xanthophylls, mammals cannot perform these biochemical reactions and need to obtain all of these carotenoids from the diet. The process of accumulation of L and Z in the eye is specific. Because of the different structures and distribution of L and Z, they may play mutual effects on visual disorders and cognition diseases. The structures of L, Z and their stereoisomers are shown in Figure 1.

## 3. The Effects on AMD

Age-related macular degeneration (AMD), the leading cause of blindness in the developed world [35,40,47], accounts for more than 50% of all blindness in the US [48]. In the UK, almost 200,000 people aged 75 years or older were visually impaired due to AMD [49]. It represents a progressive chronic disease of the central retina [50]. Owing to the sharp rise in the elderly population, the disease has brought a huge burden for the health care system and had a profound impact on the quality of life and independence of older individuals. It is estimated that by the year 2020 the number of patients with late AMD will increase more than 50% to almost three million in the USA alone [51]. Although the pathogenesis of AMD is poorly understood, oxidative stress [52] has been implicated as a major contributing factor. As L and Z are antioxidants selectively absorbed and maintained in the retina, their role in AMD has been studied extensively. AMD is a progressively degenerative disease at the central area of the retina, which results in severe visual impairment [53]. The cause of AMD is complex, and many risk factors have been implicated including age, genetics, diet and other environmental risk factors [35]. Epidemiological studies suggested that the appropriate consumption of L and Z may be associated with lower risk of AMD [54,55].

The AMD risk factors discussed above are largely unmodifiable, so it has been important for epidemiologists to focus on identifying readily modifiable risk factors that can be easily translated into public health recommendations. First and foremost, smoking has been consistent due to its oxidative burden on the body along with possible microvascular stresses. Besides, excessive light exposure has been proven to be a more controversial risk factor for AMD due to the difficulty in quantifying lifetime light exposure, so positive studies have largely concentrated on individuals with extreme levels of daily sun exposure such as fishermen who have lower rates of AMD if they use sun protection with hats and sunglasses relative to their compatriots who do not routinely employ such measures [56]. Also, animal models of AMD and cell culture studies in vitro have generally concluded that short wavelength visible light is more damaging than longer visible wavelength, a phenomenon commonly referred as the “blue light hazard” [18,57].

Increasing understanding of the pathogenesis of AMD revealed that cathepsin B and cystatin C have important functions in the catabolism of outer membranous disc of visual cells. Cathepsin B is a thiol-dependent lysosomal proteinase that can degrade collagens, connective tissue proteins, and certain native enzymes [58]. Furthermore, the expression of cathepsin B and cystatin C was significantly increased at both gene and protein levels in mice with an experimental model of AMD, which further strengthened the association of these two enzymes with the development of AMD [59]. Visual cells also secrete cystatin C, resulting in protection of the surface proteins from degradation. More recently, epidemiological evidence showed that cystatin C is associated with increased risk of developing exudative AMD [60]. Furthermore, matrix metalloproteinases (MMPs) and tissue inhibitor of metalloproteinases (TIMPs) produced by retinal pigment epithelium (RPE) cells are critically involved in maintaining the homeostasis of matrix components in the eye tissues [61]. Currently, attention has been increasingly paid to these molecular events previously not considered in the context of RPE-driven mechanisms of AMD pathogenesis. Early AMD is characterized clinically by yellowish deposits known as soft drusen accumulations and pigmentary abnormalities in the retinal pigment epithelium (RPE) and Bruch’s membrane. Whereas most of the visual loss occurs in the late stages of the disease due to one of two processes: neovascular AMD (wet AMD) and atrophic AMD (dry AMD) [47,62,63]. The recent few decades have witnessed advances in the treatment of wet AMD. Anti-angiogenic agents targeting choroidal neovascularization such as pegaptanib, bevacizumab, and ranibizumab have shown a therapeutic promise for wet AMD [64,65].

The eye is an exceptional organ because of its continuous exposure to environmental chemicals, radiation, and atmospheric oxygen. There is a general consensus that cumulative oxidative damage is responsible for aging and may, therefore, play an important role in the pathogenesis of AMD [66]. Oxidative stress may cause injury to RPE [67], Bruch membrane [68], and choroid, which are layers in the eye involved in the pathophysiology of AMD. Antioxidant strategy has been proposed and tested in the treatment of dry AMD [69]. Apart from being related to aging, recent studies showed that hydroquinone, a major prooxidant in cigarette smoke, atmospheric pollutants, sunlight exposure and diet [70,71] induced actin reorganization and bleb formation involved in sub-RPE deposits formation relevant to the pathogenesis of AMD [72]. Epidemiological evidence has shown that high-fat diet, especially consumption of saturated fats, is associated with the incidence of AMD [73]. Recent animal studies demonstrated that genetic C57BL/6 mice with hyperlipemia did not show significant RPE sediment, but the sediment was considerably increased when combined with blue light exposure, suggesting that oxidative injury caused by light exposure was necessarily required for massive formation of RPE sediment [74].

Under normal conditions, MMP-2 and TIMP-2 are expressed coordinately and maintained the homeostasis of matrix components in eye tissues. In the pathogenesis of AMD, the equilibrium between MMP-2 and TIMP-2 is disrupted, promoting the progression of the disease [75]. Present data [59] showed that these two molecules were all upregulated in ARPE-19 cells under oxidative stress. However, L/Z abolished the elevated expression of MMP-2 and TIMP-2 in H_2_O_2_-treated ARPE-19 cells, suggesting that L/Z could be beneficial for oxidative stress-involved AMD by regulating matrix homeostasis.

Genome-wide association studies (GWAS) have decisively shown that more than half of AMD risk is genetically determined by two major genetic loci, one on chromosome 1 encoding for complement factor H and the other related complement genes and another one on chromosome 10 encoding for the HTRA 1 and ARMS 2 genes, along with a large number of minor gene loci that can modestly enhance protection against or risk of development of AMD [76]. Merle et al. suggested that LIPC and LPL genes could both modify the risk for AMD and the metabolism of L and Z [77].

Since late AMD not only jeopardizes a patient’s visual function and quality of life, but also brings a tremendous socioeconomic burden, most treatment strategies are focused on addressing late AMD [78]. However, the treatment of AMD at an earlier stage might slow the progression before irreversible visual impairment occurs, which would be more effective in enhancing or maintaining visual performances [47]. A meta-analysis showed that the dietary intake of L and Z could lead to a 4% reduction in the risk of developing early AMD, as opposed to a 26% reduction for late AMD, indicating that L/Z might be more effective in reducing the risk of progression from early AMD to late AMD [79].

Current publications on the preventive and therapeutic effects of L and Z on AMD have reported encouraging results [80]. Chew [53] et al. reported that a beneficial effect of L/Z was identified when the entire study population was included, specifically, L/Z decreased the risk of progression to advanced neovascular AMD by 10%; in the secondary analysis [81], patients who had the lowest dietary intake of L/Z had a 26% decrease in the risk of disease progression. Subgroup analysis showed additional benefits. The patients who took the AREDS formulation with L/Z and no beta-carotene had 18% decrease in their risk of developing advanced AMD over the course of study compared to those who took the AREDS formulation with beta-carotene and no L/Z, as well as a 22% decrease in progression to neovascular AMD. The substitution of beta-carotene by L may further improve the formulation [82]. Nevertheless, the recently concluded Age-Related Eye Disease Study 2 (AREDS2) trial was unable to confidently demonstrate protective effects of L/Z [81,83], and whether L/Z may protect against early AMD also remains unknown. In a National Eye Institute press release [84], the primary outcome was clearly stated: “The plant-derived antioxidants L and Z also had no overall effect on AMD when added to the combination; however, they may be safer than the related antioxidant beta-carotene [85]”. Some AMD trials have found that, although macular pigment optical density (MPOD) increased after L supplementation, visual function did not show significant improvements [83,86]. This may because significant morphological changes do not adversely affect retinal function at the earlier stage, leaving little room for measurable improvement [87]. This supports the notion that early intervention might be more effective in enhancing or maintaining visual function [88].

Various observational and interventional studies have suggested that the supplementation of L and Z might reduce the risk of AMD [18,55,57,79,83,89,90,91,92,93,94,95,96,97,98,99,100,101,102,103]. Therefore, adding L and Z, along with other minerals and antioxidants to the diet in subjects with low MPOD might result in an increase in MPOD scores. Interestingly, in subjects that did not add supplementation to their diet the MPOD scores were reduced. Although diet can play a major role in the increase or decrease of MPOD, the data point to the possibility of enhancement of low MPOD through supplementation [94].

## 4. The Effects on ARC

Cataract is a clouding or opacification of the lens inside the eye that obstructs the passage of light [95]. Age-related cataract (ARC) is the leading causes of blindness and vision impairment worldwide [96,104]. It was estimated that 20 million people older than 40 years old were visually impaired due to ARC in the United States [97]. In developing countries, cataracts are the principal cause of blindness among people over 40 years of age due to improper nutrition and infectious diseases [35]. Although new therapeutic methods emerged in recent years and most ARC cases could be cured, the high treatment costs and increasing demands for therapy will challenge the long-term economic stability of health care systems [98,100]. With the rapidly aging population, ARC has brought a massive burden on health care and become an important public health issue. Thus, identifying modifiable factors available to prevent or delay the development of ARC is a crucial strategy.

The cumulative oxidation of proteins or lipids within the lens has been found to be involved in the pathogenesis of cataract, and nutritional antioxidants might protect the lens against formation of cataract [100,101,102]. Light-initiated oxidative damages are hypothesized to be the mechanism involved in ARC [103] and antioxidants might prevent or minimize oxidative damage to the lens [101,105]. As the only carotenoids present in the lens, L and its isomer Z are capable of filtering out ultraviolet light and blue light, scavenging free radicals, and thereby possibly decreasing light-induced oxidative damage to the lens, which indicates that they may play a protective role in the prevention of cataract [18,57].

Risk factors for cataract development include increasing age, diabetes [106], smoking [107], alcohol use, trauma, and prolonged exposure to UV light [108]. A longitudinal study has shown that plasma Z reduces the risk of cataract [109]. Xanthophylls, in particular Z, could attenuate photochemical damage by filtering high-energy short-wavelength light [110]. In addition, they serve to protect the lens from oxidative damage by scavenging reactive oxygen species (ROS), indicating that these carotenoids may play a potentially important role in the prevention of ARC [111]. However, others failed to find such association or the results regarding certain subtypes of ARC were inconsistent [25,112]. Besides, a meta-analysis reported that no significant protective effects were found for each of these carotenoids against either cortical cataract or posterior subcapsular cataract, except a borderline significant association between blood L and subcapsular cataract [113]. Chew et al. failed to prove a protective effect for β-carotene or for L and Z with respect to cataracts or cognitive function [83,107].

The difference in associations between cataract subtypes and serum L and Z was probably due to the distinct pathogenesis for each type of ARC [114]. With increasing age, a lower percentage of reduced glutathione can reach the lens nucleus, which makes the nucleus become less able to repair oxidative damage [115]. In contrast, glutathione levels in the outer cortex of the lens remain at high levels, even in nuclear cataracts [116]. Therefore, nuclear cataract may be more prone to a significant association with serum L and Z. In addition, Gale [117] et al. have found that risk of posterior subcapsular cataract was lowest in those with higher concentration of L.; however, subcapsular cataract is least common among these three main types of ARC and further studies are needed to confirm such finding [118]. Besides, Liu [113] et al. demonstrated that increased blood concentrations of L and Z might be associated with a reduced risk of nuclear cataract. However, there is insufficient evidence to support a significantly inverse relationship between blood L or Z level and risk of other subtypes of ARC [113].

Evidence has emerged suggesting that healthy lifestyle and proper nutrition may have a beneficial effect on the onset of cataracts [119]. Numerous epidemiological studies have investigated the relationship between dietary intake and blood levels of L and Z and the risk of ARC [120]. Because the accuracy of dietary intake measurements is greatly influenced by the different dietary assessment methods across the studies and the individual differences in utilization and absorption, blood concentrations appears to be a stronger predictor of nutritional status [121].

Therefore, dietary L and Z intake might be associated with a reduced risk of ARC, especially nuclear cataract in a dose-response manner, indicating a beneficial effect of L and Z in ARC prevention [122].

## 5. The Effects on Cognitive Function

The relationship between L, a dietary xanthophyll carotenoid, and visual and cognitive health is particularly compelling because L is taken up selectively into eye and brain tissue [10,123,124]. In part, the beneficial effects of L are thought to be attributable to its antioxidant and anti-inflammatory properties. Given that the eye is an extension of the neural system, L is increasingly recognized as having a role in cognitive function [26]. In pediatric brains, the relative contribution of L to the total carotenoids is twice that found in adults, accounting for more than half the concentration of total carotenoids. The greater proportion of L in the pediatric brain suggests a need for L during neural development as well [125]. Apart from cognitive function relationships with macular pigment there is less evidence for a relationship between Z and cognition. A number of recent studies evinced a possible role for L and Z in cognitive function [123,126,127]. For instance, one of the investigations conducted as part of the Irish Longitudinal Study on Aging determined that older adults with higher macular pigment optical density (MPOD) had better results in various indices of cognitive function compared to those with lower MPOD [126]. Furthermore, several studies have shown cognitive impairment to be related to age-related eye diseases (AREDs) [128,129], suggesting that similar factors may be involved. These observations are in line with the view that vision and cognition are not easily separable. The rationale supporting a role for L in cognitive function is based on the following observations: (1) L is the predominant carotenoid in human brain tissue in early as well as late life [123,124]; (2) primate retinal L concentrations, i.e., macular pigment density, are related to brain L concentrations [130]; (3) macular pigment density is related to cognitive function in adults [126,127]; and (4) L supplementation in adults improves cognitive function [131].

### 5.1. The Effects on Infancy Cognition Function

Environmental enrichment, as would occur with visual cues, has long been investigated as an influence on brain structure and function. Morphological and functional effects elicited by environmental enrichment at the neuronal level have been reported to be accompanied by improvements in cognitive performance [132].

The retina and brain are especially in need of antioxidants because of their high metabolic rates, and the human newborn brain has a relative deficiency of endogenous antioxidant enzymes [133]. Nevertheless, the relative contribution of L to total carotenoids was approximately twice that found in adults (59% vs. 31%, respectively) [123,124], indicating a possible additional role of L in early neural development. Optimal visual performance in early life could influence brain development, which is rapid in the first year [134]. Besides, analyses of the various cognitive scores revealed no significant relationship between MPOD levels and cognition. Zimmerman et al. suggested that MPOD can be used as a biomarker in order to determine the amount of L and Z that people have in their brain tissue in its infancy [135]. Vishwanathan et al. further identified that biochemically measured concentrations of L and possibly Z in the macula are a biomarker of brain L and Z status in primates. Therefore, an integrated measure of total MPOD may be a useful tool in evaluating the role of xanthophylls in cognitive function [130].

### 5.2. The Effects on Adult Cognition Function

Among the carotenoids, L and Z are the only two that cross the blood-retina barrier to form macular pigment in the eye [38], and L is the dominant carotenoid in human brain tissue [123,124,130,136,137,138]. And only L was consistently associated with a wide range of cognitive measures that included executive function, language, learning, and memory, which are all associated with specific brain regions [123]. Besides, in a double-blinded, placebo-controlled trial of women who received L supplementation (12 mg/d), docosahexaenoic acid supplementation (800 mg/d), or a combination of the two for 4 months, verbal fluency scores improved significantly in all three treatment groups. Memory scores and rates of learning improved significantly in the combined treatment group, who also displayed a trend toward more efficient learning [131]. Terry et al. suggested that individuals with less L may have compensatory neural mechanisms to help them to engage in learning and recalling processes [139]. Taken together, these observations suggested that L could influence cognitive function. Hoffmann [140] et al. found that supplementation of Z promoted a better long term delayed memory.

How (and really if) they influence brain function, however, is less clear. One possibility is simply protection from the accumulated effects of oxidative and inflammatory stress [141]. Data linking reduced MPOD to dementia [142] and cognitive impairment [143] is consistent with that possibility. Another possibility, more relevant to younger individuals and palliative approaches, is a direct improvement by some type of local interaction with neural cells (the so-called neural efficiency hypothesis) [144]. Besides, it has also been suggested that the carotenoids may play a beneficial role by enhancing gap junctional communication in the brain [125]. Furthermore, the visual benefits of MP are not restricted to the effects of its optical properties, reflected in a growing body of evidence that the macular carotenoids may have a favorable effect on neuronal processing [145]. These carotenoids have been shown to improve communication through cell-to-cell channels, modulate the dynamic instability of microtubules (structural units of neurons), and prevent degradation of synaptic vesicle proteins [146,147].

### 5.3. The Effects on Alzheimer’s Disease (AD)

Alzheimer’s disease (AD) is an age-related neurodegenerative disease characterized by the accumulation of amyloid plaques and neurofibrillary tangles in the brain [148]. It is the most common form of dementia. Although the cause of AD remains unclear, genetic predisposition and environmental factors are thought to initiate a pathophysiologic cascade, leading to AD pathology and dementia. Accumulating evidence suggests that oxidative stress plays an important role in disease pathogenesis [149]. Free radical species produced during oxidative stress are suspected to mediate protein oxidation, lipid peroxidation, and DNA and RNA oxidation in multiple brain regions [150]. Devore [136] et al. have shown that antioxidant-rich food reduces the risk of AD by inhibiting oxidative stress. Besides, Lindbergh et al. demonstrated that L and Z may benefit cognitive function in older adults by increasing neurobiological efficiency in brain regions at risk for age-related deterioration [137]. Recent preliminary data have shown that L may influence the differentiation of pluripotent neural stem cells [138]. However, opinions vary. Among older persons with AMD, oral supplementation with LCPUFAs or L/Z had no statistically significant effect on cognitive function [107]. Cognitive dysfunction is a complex process. More research to be conducted is still needed to verify the reliability of the results.

## 6. The Consumption and Edible Safety of Lutein

L is present in natural foods and does not need to be added in a balanced diet. L is beneficial to human health and is closely related to the occurrence of some diseases, such as AMD, ARC etc. When dietary intake is insufficient, the appropriate dosage of L may have a positive effect on preventing the occurrence of related diseases. For people aged, 18–79 year in many countries, the lowest dietary intake of L is 0.67 mg/d [151], the highest is 6.88 mg/d [152], and the normal intake level always lies in 1.10–4.25 mg/d [120,153,154,155,156,157,158,159,160]. According to the limited dietary survey data in China, in people above 30 years of age, the minimum dietary intake of L is between 1.48, 10.20 mg/d [160,161] and the maximum is between 2.94 [162] and 7.77 mg/d [163].

### 6.1. The Intervention of Lutein in AMD Study

L intervention study showed that L supplementation can improve the L level in patients with AMD, and to a certain extent, can improve a visual function index. The improvement of visual function generally in L intervention for a long time is effective. For example, the focus of its multifocal electroretinogram response amplitude increased significantly after taking L 10 mg/d per day for the early and middle stage AMD patients [164]. The MPOD of male patients with AMD was increased by 36% after one year of taking L 10 mg/d. And visual acuity, contrast sensitivity and glare recovery time were improved significantly [165]. The TOZAL [166] study also found that visual function was improved when daily use of L 8 mg for more than 6 months for patients with dry AMD. In general, in the intervention study of L in the AMD population, the significant dose range of L intervention was 2.5 [167]–20 mg [168], and the lowest effective dose was 2.5 mg/d. In addition, the duration of intervention was closely related to the intervention dose.

### 6.2. Intervention Doses in Lutein and ARC Studies

The results of L intake showed that L intake of 6–10 mg/d can reduce the possibility of cataract surgery 20–50% [169]. When L/Z intake was 2.4 mg/d, the risk of core lens opacity was reduced significantly. In a limited study on the intervention of L in ARC, by double-blind of intervention study of ARC patients, Olmedilla [170] et al. found that the level of serum L, visual acuity and glare sensitivity improved significantly. In this study, the intervention dose of L was 6.42 mg/d. These results indicated that L has a positive effect on the prevention of cataract [171]. In the ARC study, the effective dose of L was 2.4–6.42 mg/d [93,170,172]. Nevertheless, the intervention of L on cognitive function has not been reported.

### 6.3. Safe Consumption of Lutein

According to the toxicological evaluation of L food safety, the ADI value of EFSA is 1 mg/(kg·d) [173], JECFA 2 mg/(kg·d) [174]. The former is much safer. That is to say, it is safe for 60 kg adults taking in L at 60 mg/d. EFSA panel [175] pointed out that the content of L in infant formula milk power and food is not more than 250 μg/L. The results showed that the serum L level of smokers was significantly lower than that of non-smokers [176]. Smokers are at a state of oxidative stress for a long time and they have a higher risk of disease. L can prevent the occurrence of related diseases by anti-oxidation [177]. Nevertheless, the study [176] found that smokers who take high doses of L supplement have an increased risk of lung cancer. Therefore, caution should be given to smokers with higher doses of L (>10 mg/d) supplements. Excessive intake of L can cause yellowing of the skin. As in the continuous taking higher doses of L (≥15 mg/d) after 2–5 months, skin stained yellow. But this change is reversible and it will not damage the organs. Yellow skin will die away after stopping taking L for a period of time [37,178]. Because L is a fat soluble compounds and it can accumulate in the body. The excessive consumption of L can increase its concentration in plasma and skin, leading to yellow skin [179]. All in all, L is a component of human food. Its safety has been proved by the toxicological evaluation of food safety, authoritative institutions at home and abroad, the results of the study of population and clinical intervention. It is safe to human body in a certain range of use (≤60 mg/d).

## 7. Conclusions and Future Directions

L and Z are selectively concentrated in the different zone of eyes. Because of their physical and chemical properties, the direct biological effects of L are mainly manifested in antioxidation, filtering blue light and the formation of retinal MPs. Thus, it reduces damage to the retina. In addition, L and Z distributed in the macular area may play a protective role in retinal cells, such as preventing the occurrence of AMD, ARC and cognitive function etc. The AREDS 2 project completed in the United States in 2013 for 4203 AMD patients with a combination of L for 5 years was negative [180]. The study should focus on the prevention of high risk groups to avoid the occurrence of AMD or ARC, rather treatment. At the same time, the negative results of the AREDS 2 study could not be understood as the negative result of the biological function of L in the prevention of disease. Besides, the biological effect of L is not proportional to the food consumption. Long term intake of high doses of L by smokers are associated with increased risk of lung cancer [176]. The best biological effects can be achieved only at the appropriate dose. As mentioned above, L supplements, especially for smokers, are not consumed more than 6 mg/d. Based on the analysis of the present research data, it is suggested that L can play a role in the regulation of cell signal transduction pathway through its antioxidant, anti-inflammatory effects as well as the conduction velocity of nerve cells. And the intervention dose of L on cognitive function remains to be further studied.

How to set the amount of L supplements and the use of methods, in order to achieve the purpose of avoiding the occurrence of yellow skin? Studies have shown that in the human body, it takes 14–16 h for plasma concentration of L to reach its peak following intake [181,182]. About the half-life in blood and cleared time difference between the results of study, some studies suggest that plasma L has a half-life of about 76 days [183]; studies also have shown that L is cleared in only 528 h (that is 22 days) [184]. If the L in plasma reached the effective concentration, using the method of intermittent treatment L supplements to maintain its lowest effective dose range, it would be more beneficial than continuous supplement once a day. However, it is necessary to determine the kinetics of blood metabolism and the relationship between time and dose effect for a brief time and dose.

In summary, the aim of study is to explore the edible quantity and edible method which can achieve the best biological effect and can be used for a long time without adverse effect, such as for skin yellowing and smokers. Based on the results of the present study, the recommended dietary intake of L supplements does not exceed 6 mg/d, methods for the consumption of L supplements can be tried in a discontinuous manner. Further research is needed to provide sufficient evidence.

## Abbreviations

The following abbreviations are used in this manuscript:

L	lutein
Z	zeaxanthin
MZ	meso-zeaxanthin
MP	macular pigment
AMD	age-related Macular degeneration
ARC	age-related Cataract
AD	Alzheimer’s disease
MPOD	macular pigment optical density
ADI	acceptable daily intake
mg/d	mg/day
kg·d	kg·day
